# Need for including Hand Eye Coordination and Hand Function Training in the Management of Adhesive Capsulitis – A non-randomized control trial

**DOI:** 10.12669/pjms.38.3.5153

**Published:** 2022

**Authors:** Senthil Kumar B, Subbaiah S, Arunachalam Ramachandran

**Affiliations:** 1 Senthil Kumar B., (Ph.D.), MPT, MIAP, Ph.D. Scholar. Saveetha College of Physiotherapy, Saveetha Institute of Medical and Technical Sciences, Principal, UCA College of Physiotherapy, Chennai, India; 2 Dr. Subbaiah S, MBBS, MS, D. Ortho, PG Cert (Hand Surgery) Professor & Head of the Orthopaedics, Department of Orthopaedics, Saveetha Institute of Medical and Technical Sciences, Chennai, India; 3 Dr. Arunachalam Ramachandran., MPT, MIAP, PhD. Principal & Professor, College of Physiotherapy, Madhav University, Rajasthan, India

**Keywords:** Adhesive capsulitis, Disability, Hand-Eye Coordination, Hand Function, Shoulder Range of motion, Quality of life

## Abstract

**Objectives:**

The objective of the study was to identify the effect of Maitland mobilization with hand-eye coordination and hand function exercises in the prognosis of adhesive capsulitis.

**Methods:**

This non-randomized control trial was done with 40 patients with adhesive capsulitis referred to the OPD at UCA College of Physiotherapy, Chennai. The study was performed for 8 months duration from August 2018 to March 2019. After providing a sufficient explanation of the procedure, the patients were divided into Group-A and Group-B. Group-A were allocated to Maitland group (n=20) (MG) and Group-B, were assigned to the Maitland, Hand-eye coordination and Hand Function exercises group (n=20) (MHG), respectively. We measured Quality of life using SF36 to know whether these patients had poor QOL compared to normative. Each patient underwent testing before the experiment to evaluate the range of motion of the shoulder (Abduction and External rotation) measured using a 180º goniometer, Functional Disability assessed using SPADI index. Statistical analysis was applied using SPSS version 20.0. Before the study, data normality was tested. A parametric test was used to compare pre-and post-intervention data in each Group-And also compare the MG vs. MHG. An *α* < 0.05 is the level of significance in all analyses.

**Results:**

A total of 40 subjects accounted for the study results. Their quality of life was significantly lower from the normative values. Both groups were homogenous at baseline with no significant difference between the ROM and SPADI scores. Both groups showed a significant improvement in ER, abduction ROM and SPADI scores, however the between group posttest analysis revealed that the Group-B subjects progressed significantly better.

**Conclusions:**

The study concluded that the Maitland mobilization and hand-eye coordination and hand function exercises are an effective tool in improving shoulder abduction, external rotation and shoulder functions.

## INTRODUCTION

Adhesive capsulitis is one of the most prevalent condition causing shoulder pain and disability in the general population.^1^ Onset is insidious and has painful restrictions on the shoulder movements, and there is a severe global restriction in the glenohumeral joint.^2^ It occurs about 2% to 5% in the general population and mainly occurs at 40-65 years.^3^

Pain in and around the shoulder, limited range of motion (at first, external rotation and abduction followed by the entire shoulder), altered kinematics of the shoulder (abnormal scapulohumeral rhythm), wasting or muscle weakness, absenteeism at work, and inability to perform leisure activities are all common clinical symptoms.^4^

Altered shoulder kinematics, including limited external rotation of the humerus, restricted posterior tipping and upward rotation of the scapula during arm elevation, and limited range of shoulder motion with muscle weakness are predominant.^5^ The exact pathomechanics of adhesive capsulitis is unclear, but perhaps the most generally recognized hypothesis states that inflammation occurs in the shoulder joint capsule and the synovial fluid^6^ This inflammation causes reactive fibrosis and creates adhesion of the synovial lining in the joint, restricting joint motion. First, there is an inflammation of the capsule that causes pain, the fibrosis of the capsule, and adhesions that lead to decreased range of motion.^7^

Hand-eye coordination may need the brain to integrate the visual inputs with the continuous change in head, eye and arm positions.^8^ Lack of coordinated voluntary hand movements is more common in the population with shoulder disorders. Regular hand-eye coordination involves the synergistic function of several sensorimotor systems like vestibular, visual, proprioception, and arms control. It also includes several closely regulated neurophysiological processing loops involving different neurotransmitter systems.^9^

Conservative measures such as anti-inflammatory drugs, intra-articular corticosteroids, and Physiotherapy are used to treat adhesive capsulitis.^10^ Conventional Physiotherapy measures include heat, ultrasound therapy, active range of motion exercises, and passive mobilization exercises. Various mobilization techniques are followed, which includes Maitland. Mobilization and Manipulation restored the pain-free state and regular use of the upper extremity.^11^

Electrotherapy can help with pain management in the short term. Continuous passive motion is recommended for pain relief for a short time but not for increasing range of motion or function. Deep heating could be used to relieve pain and improve the range of motion. Ultrasound is recommended for pain relief, improving range of motion, and not improving function.^11^-^14^

Maitland mobilization is the standard technique that is used in the rehabilitation of shoulder disorders. It includes accessory oscillatory movements to treat stiffness and improve motion range.^15^ Grades are used to relieve pain and improve motion in the joint. Studies showed that Maitland mobilization is more effective than the conventional exercise program in adhesive capsulitis.^16^

Hand-eye coordination exercises promote the synergistic functioning of the perception and the motor system^17^. It is the essential element in movement forums and improvement of motor skills to improve the skill development. It also helps to increase the overall motor skill proficiency and facilitate participation.^18^ Hand Function Exercises improves Hand Function. It involves the synergistic functioning of the perception and motor systems.^19^

Adhesive capsulitis is one of the common conditions which exhibits shoulder pain and stiffness, and this is associated with the muscles, ligaments, cartilages, and capsules. A deep relationship theoretically exists on the upper limb functions regarding the shoulder and the wrist and hand.^20^ Neurological patients who have the involvement of the upper limb were used to train with wrist and finger motions concerning the shoulder. However, there are no such studies in musculoskeletal dysfunctions. This study aimed to identify the effect of Maitland mobilization with hand-eye coordination exercises and Hand function training exercises on various outcomes in adhesive capsulitis.

## METHODS

The non-randomized control trial includes participants with adhesive capsulitis referred to the OPD at UCA College of Physiotherapy, Chennai. The study was performed for eight months duration from August 2018 to March 2019. Those who visited were screened for age, gender, affected shoulder, duration of pain, previous management, medical history, disability, pain, and functional disability. The inclusion criteria are patients with pain lasting at least three months, a 50% restriction in range-of-motion (abduction and external rotation) compared to the non-affected shoulder, 40 to 60 years old, no previous surgery to the affected shoulder, no manipulations under anaesthesia to the affected shoulder.^21^ Patients with rheumatoid arthritis, osteoporosis, surgical fixation in the affected limb, radiating pain due to cervical conditions, neurological damage due to stroke or Parkinsonism’s were not included. As this was a time bound study sample size was not estimated. The same is justified in the methodology. Protocol of the trial was not registered

After providing a sufficient explanation of the study procedure, the patients who volunteered to participate in this study were provided with a written consent form. Following the Declaration of Helsinki, this study was approved by the Ethics Committee of Saveetha Institute of Medical and Technical Sciences, Chennai (vide Certificate No. 003/04/2018/IEC/SMCH dated 12/04/2018). Once the patients signed it, they were all randomly assigned to one of two therapists, a sealed box contains a card prepared with A or B, and the patients choose one card. Patients with card A were allocated to Maitland group (n=20) (MG), and with card B, they were assigned to the Maitland, Hand-eye coordination and Hand Function exercises group (n=20) (MHG), respectively. Each patient underwent testing before the experiment to evaluate range of motion of the shoulder (Abduction and External rotation), Functional disability and Quality of life which was measured using 180º goniometer, SPADI index and SF36 respectively.

Once the treatment began, warm-up of the shoulder was given by using a heating pad for 15 minutes, followed by Maitland mobilizations. Each joint mobilization was performed by the senior therapist who has experienced more than ten years in the mobilization techniques. Patients received 12 therapy sessions three times in a week for four weeks. All the patient’s outcomes were evaluated following four weeks of therapy. The patients were recommended to use their upper limbs with all the possible daily activities; they have all received a set of home exercises. Patients in both groups received Maitland mobilization three times a week for 30 minutes. Maitland mobilization was performed to the affected shoulder as described by Maitland, and the patient is in a supine lying position. The humeral head was moved to the resting position of 40º abduction while maintaining in this position 10 to 15 repetitions of shoulder glides were applied^22^. Glides include shoulder caudal glide, posterior-anterior glide, and antero-posterior glide. Oscillatory movements at a rate of 2—3 glides in a second for 30 seconds of each glide and applied for five sets^16^. Hand-eye coordination exercises include repeated juggling of the balls, catching the ball, pegboard exercises, ball wall toss, and tossing ball and catching. Hand Function Exercises include squeezing balls of different sizes, various grasp movements, therapeutic putty exercises, wrist curls, finger spreading and rolling movements. These exercises were demonstrated to the participants (MHG group) and advised them to do 30 minutes for three days a week.

Statistical analysis was done using SPSS version 26.0. Parametric analysis (Independent and paired t test) was used for the ROM of abduction and external rotation and non-parametric analysis (Wilcoxon and Man Witney test) was performed for SPADI. Before the study, data normality was tested. An *α* < 0.05 is the level of significance in all analyses.

## RESULTS

**Fig.1:**
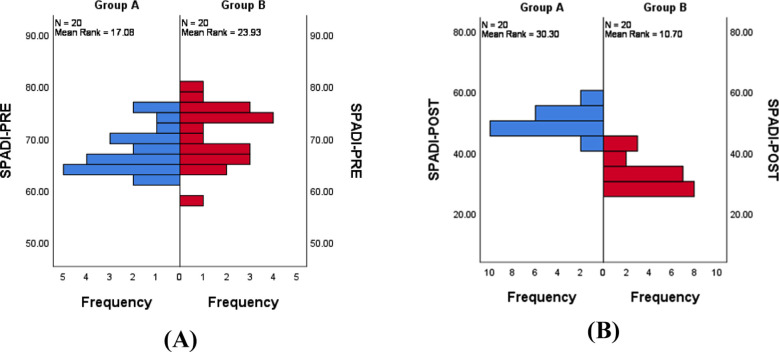
Between Group-Analyses of pre-test (A) and post-test (B) SPADI scores.

A total of 40 subjects accounted for the study results as there were no drop-outs. The study used three primary outcome measures and one secondary outcome measure. The abduction and external ROM along with the SPADI scale were the primary outcome measures and the SF-36 was taken to know whether the quality of life differed for the study subjects compared to the normal. The demographic data of the subjects are provided in the Table-I. The quality of life of the study samples were significantly lower from the normative values which is shown in the Table-II. Within Group-Analysis was performed to know whether the subjects in both group improved with the intervention. The analysis for abduction and external rotation are provided in Table-III. In the SPADI analysis it is obvious that there was a significant prognosis in both the groups with Z value of -3.927 and p <0.001 and z value of -3.925 and p < 0.001 for Group-A and B respectively. The between Group-Analysis of abduction and external rotation showed that there was no difference between the pre-test values which proves the baseline homogeneity. The between group post-test analysis showed that Group-B showed a statistically significant improvement than Group-A as displayed in the Table-IV. There was a similar trend in SPADI analysis as well with Group-B performing better than Group-A with p value of <0.001. The performance of SPADI is displayed in Fig.1.

**Table I:** Demographic data (n=40).

Criteria	N (%)
Sex	
Men	12 (30)
Women	28 (70)
Age, mean (SD) (year)	52(03)
Dominant hand, n (%)	
Right	36(90)
Left	4(10)
Affected limb, n (%)	
Right	10 (25)
Left	30 (75)
Chronicity	
Less than 1 month	21 (52)
1-2 months	07(18)
2-3 months	12(30)

**Table II:** Comparison of SF-36 different domains scores in patients suffering Adhesive capsulitis with normal population.

SF-36 domains	Mean	SD	Normal population mean	P value
Physical function	62.4	21.9	55	0.001
Role physical	27	22.5	50	<0.001
Body pain	35.8	19.6	48	<0.001
General health	52.1	17.1	55	0.005
Vitality	49.2	13.2	63	<0.001
Social function	62.2	21.9	66	0.001
Role emotion	48.2	37.2	63	<0.001
Mental health	53.2	18.2	63	<0.001
Physical component summary	36	8	52	<0.001
Mental component summary	43.2	10.8	64	0.001

**Table III:** Within Group-Analysis of Abduction and external rotation.

	Groups	M	SD	SE	t	P
Abduction	GROUP-A-PRE	64.85	6.40	1.431	-39.05	0.00
	GROUP-A-POST	91	6.19	1.386		
	GROUP-B-PRE	64.5	6.40	1.43	-28.33	0.00
	GROUP-B-POST	118	10.9	2.44		
External Rotation	GROUP-A-PRE	44.65	6.86	1.536	-15.07	0.00
	GROUP-A-POST	58.20	4.44	2..99		
	GROUP-B-PRE	45.05	6.64	1.48	-15.858	0.00
	GROUP-B-POST	75.75	7.12	1.59		

**Table IV:** Between Group-Analysis of Abduction and external rotation.

	Groups	N	Mean	SD	SE	F	p
Abduction-Pre	Group-A	20	64.85	6.40	1.43	.002	.968
	Group-B	20	64.00	6.40	1.43		
Abduction-Post	Group-A	20	91	6.19	1.38	8.30	.006
	Group-B	20	118	10.9	2.44		
ER-ROM-Pre	Group-A	20	44.65	6.86	1.536	5.036	.031
	Group-B	20	45.05	6.64	1.48		
ER-ROM-Post	Group-A	20	58.20	4.44	.993	5.036	.031
	Group-B	20	75.75	7.12	1.592		

**Fig.2:**
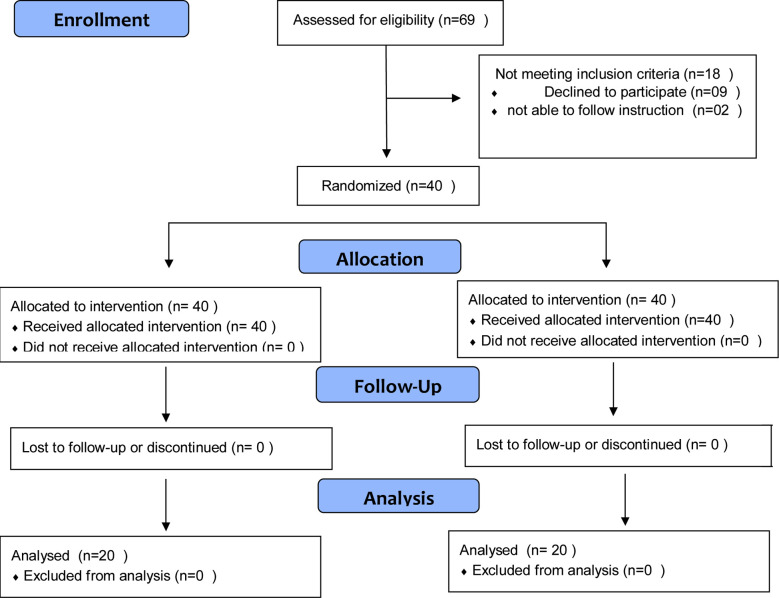
CONSORT Flow Diagram of the study.

## DISCUSSION

The study aims to find out the effect of Maitland mobilization with hand-eye coordination and hand function exercises on various outcomes in adhesive capsulitis. Adhesive capsulitis is characterized by pain and decreased range of motion in the shoulder, which adversely affect the whole upper limbs.^23^ Management of the adhesive capsulitis focuses on improving shoulder dysfunctions, whereas the other parts are often not considered. This study tries to identify by adding hand-eye coordination and hand function exercises on adhesive capsulitis. Some studies suggest that shoulder disorder has some effect on hand functions.^24^

Published literature shows that mobilization is effective in adhesive capsulitis, and they also evaluated that Maitland mobilization improves the range of motion.^25^ Traction at variable amplitude applied at a low velocity decreases the intra-articular compressive forces, which removes the distension of the peri-articular tissues.^26^, ^27^ These non-stretch motions promote the synovial fluid motions and improve the nutrition to the cartilage and reduce pain^16^. Repeated oscillatory stimulation blocks the nociceptive pathways at the spinal cord level by inhibiting the perception of pain by the mechanoreceptors.^28^

Passive oscillatory joint movements are performed on the glenohumeral joint, helps improve shoulder range of motion and reduce pain.^25^ It was suggested that functional disability was reduced in relation to the changes of perception of pain and improved range of motion. Mobilization also aids in improving daily activities, pain-specific tasks, and lifting actions.^28^

Hand function has been shown to correlate with the strength of the upper extremity, and it is used as an objective measurement for the upper extremity functions. So, the addition of hand exercises helps improve shoulder function and upper limb activities.^29^ Hand-eye coordination exercises affect fine motor skills in addition to gross motor functions. These exercises enhance the precision of the hand function in association with the shoulder and arm muscles, and it was observed that without any remarkable changes in either latency or duration of an eye, head, or hand movements.^30^

After analysis, this study concluded that Maitland mobilization, along with the hand-eye coordination and hand function exercises, was much more superior to the Maitland exercises alone; however, the home exercises were included for both groups. There was significance at *p <* 0.05 with a 95% confidence interval between the groups for Range of motion (Abduction and External rotation), SPADI and SF36. So, this study allowed the rejection of the null hypothesis. Thus, there is strong support for the use of hand-eye coordination and hand function exercises in addition to the shoulder mobilization will improve more upper limb functions than only concentrating on the shoulder alone. Future studies can also compare different levels of Maitland mobilization in this regard. 31

### Limitations of the study

Limitations of the study noted that as the Maitland and Hand-eye coordination and hand function exercises results improved in the functional, no follow-up is made in the long term. The sample size was not large enough to generalize the study to other populations. The application of mobilization techniques varies when the patient starts improving, and future studies can also include different types of shoulder conditions to identify the hand-eye coordination exercises.

## CONCLUSION

This study concludes that adding hand eye coordination and hand function training with Maitland manipulation in the management of adhesive capsulitis yield better results in improving abduction ROM, external rotation ROM and decreasing the pain and disability. The major clinical implication from the study is inclusion of hand eye coordination and hand function training exercises in the conventional management of Physiotherapist in adhesive capsulitis. Future studies should include EMG analysis of the upper limb in analyzing the patterns of movement that helps in shoulder recovery when hand function and coordination are trained.

### Author’s contribution:

**SKB & SS:** Contributed to the study concept and design.

**AR & SS:** Have helped in data acquisition.

**SKB:** Did the analysis of the data, prepared the first draft of the paper and revised the manuscript. All authors read and approved the final manuscripts and are responsible for the accuracy and integrity of the work.
